# Knowledge, Attitude and Practice in Anxiety, Pain and Medical Emergency Management: A National Survey on 232 Italian Dental Students

**DOI:** 10.1111/eje.70012

**Published:** 2025-07-11

**Authors:** Niccolò Giuseppe Armogida, Luigi Esposito, Gianrico Spagnuolo, Mariangela Cernera, Carlo Rengo, Luca Signorini, Antonino Fiorino

**Affiliations:** ^1^ Department of Neuroscience, Reproductive Sciences and Dentistry Federico II University of Naples Naples Italy; ^2^ Saint Camillus International University of Health Sciences Rome Italy

**Keywords:** anxiety, dental students, knowledge, medical emergencies, pain

## Abstract

**Background:**

Anxiety, pain and medical emergencies are crucial concepts in the dental field. Dental students need training, but little is known of their competence on these topics.

**Aim:**

The aim of this study was to assess the current state of Italian dental students' preparation for, attitudes to, and perceptions of medical emergency, anxiety and pain management.

**Materials and Methods:**

A cross‐sectional study was conducted on Italian dental students with the collaboration of the Italian Association of Dentistry Students. Students were sent a questionnaire, the contents of which were as follows: Part I (demographic information); Part II (22 questions: 12 on Knowledge, 8 on Attitude and 2 on Practice, relating to three domains: treatment of anxiety, pain and medical emergencies). The Knowledge section consisted of 12 multiple‐choice questions, each with five answer options, only one of which was correct; a score of 1 was assigned for each correct answer and 0 for incorrect answers, resulting in a total score ranging from 0 to 12. The Attitude section included 8 questions, each with five possible answers scored from 0 to 4, for a total score range of 0 to 32. The Practice section comprised 2 questions with three response options, evaluated with scores from 0 to 1, yielding a total possible score between 0 and 2.

**Results:**

From the 38 participating universities, a total of 232 eligible questionnaires were received with an average of 6.11 questionnaires per university site. Knowledge section: The mean score was 3.51 ± 1.79 points without geographical differences. For the *anxiety domain*, the mean total score was 0.54 ± 0.66 points. A geographical difference (p‐value: 0.049) was observed between the North (0.41 ± 0.59) and the South of Italy (0.65 ± 0.67). For the *pain domain*, the mean result was 1.31 ± 0.98 points. For the *medical emergency domain*, the mean result was 1.65 ± 1.01 points. No geographical differences were found in the pain and emergency domains. Attitude section: The mean total score was 17.45 ± 3.68 without geographical differences. Practice section: 53.45% of students stated that they had never taken courses in anxiety management. Regarding emergencies, 25.43% had never attended BLS courses. No geographical differences were found.

**Conclusion:**

There is a clear need to improve the effectiveness of university training to guarantee the skills to identify/prevent medical complications related to anxiety and pain in clinical practice.

## 1 Introduction

Anxiety, pain and medical emergencies are crucial concepts in the dental field. Understanding the knowledge, attitude and practices related to these aspects is essential to improve clinical management and ensure quality dental care.

Anxiety is a physiological and psychological reaction that occurs in response to a perceived stressful or threatening situation. Dental anxiety can have a significant impact on patients' oral health, negatively affecting adherence to dental care [1], quality of life and psychological wellbeing [2, 3]. Previous research has indicated that dental anxiety often leads to appointment cancellations, missed visits, or late arrivals [4].

Several studies have shown that between 10% and 35% of the population suffers from medium or high‐degree dental anxiety [5]. One study reported that 12% of the population suffers from odontophobia, a pathological condition of psychiatric concern. Dentally anxious patients often experience poorer dental health due to prolonged delays in seeking dental care, resulting in complications and the need for more complex and intricate treatment [6, 7]. Managing patients with dental fear is a significant source of stress for many dentists [8], which can lower the quality of care provided.

Pain, alongside anxiety, is central to dental patient experience andis defined as an unpleasant sensory experience characterised by a significant emotional component. Numerous studies have shown a close correlation between anxiety and pain, where the increase in anxiety can intensify the perception of pain and vice versa. Studies investigating patient responses to surgical procedures have found that individuals with higher levels of preoperative anxiety and greater expectations of pain tend to experience more postoperative pain [9]. The close relationship between anxiety and pain is well‐established, with anxiety even being considered a predictor of pain [10, 11]. Understanding this connection is essential for adopting a comprehensive and personalised therapeutic approach to pain management in dental patients [2, 3].

Not only an experienced dentist, but also a newly graduated one, should be able to manage the patient's mental state and pain, as well as be prepared to deal with various emergency scenarios. Medical emergencies are an inevitable reality in dental practice, and the development of specific protocols is a necessity [12]. Not many studies in the literature have investigated this, but a percentage ranging from 36% to 76% of dentists have had to face at least one medical emergency during their career. Of these dentists, about 10% to 16% have been required to provide urgent care for a serious cardiac event [13]. In some European countries, the average annual incidence of cardiac arrest ranges from 36.8 to 39.7 per 100 000 inhabitants [14], and the number of reported out‐of‐hospital cardiac arrests in Europe has increased in the last two decades [15]. Although medical emergencies are not a frequent occurrence in the dental clinic, their incidence should not be considered insignificant. For this reason, dentists must be skilled in the initial management of the main urgent/emergency medical situations.

In Europe, since 1972, the Association for Dental Education in Europe (ADEE) has standardised the profile of competencies, knowledge and skills that a recent graduate in dentistry must possess [16]. Regarding anxiety and pain, the ADEE emphasises that dentists must possess skills in recognising, classifying and treating dental fear/anxiety and orofacial (including temporomandibular joint—TMJ) pain in the pre‐, trans‐ and post‐operative phases. Reference is also made to postgraduate training courses to develop and enhance dentists' competencies (Domains IV and VI).

Similarly, in the context of medical emergencies, dental graduates must be able to assess clinical risk and appropriately manage a broad range of conditions, including cardiovascular events, respiratory distress, airway obstruction, metabolic imbalances (e.g., hypoglycaemia and hyperglycaemia), allergic reactions, vasovagal syncope, seizures, haemorrhage, altered consciousness and other acute conditions. These requirements underscore the importance of integrating the management of anxiety, pain and medical emergencies into both undergraduate and postgraduate dental education, as they are interrelated aspects of clinical dental care.

In Italy, dental education is currently organised as a six‐year single‐cycle degree program in Dentistry and Dental Prosthodontics. This course of study became formally independent from Medicine in 1985, following the Ministerial Decree of January 30, 1985. Prior to this reform, the qualification to practise dentistry was obtained through a degree in Medicine and Surgery, followed by a three‐year specialisation in Odontostomatology.

Admission to dental programs is regulated by a national entrance examination. Although all dental schools are accredited by the Ministry of University and Research (MUR), there is no centralised system to ensure uniformity in educational outcomes. Each institution maintains autonomy over curricular content, teaching methods and student assessment, resulting in variability across programs. Until recently, graduates were also required to pass a national licensing examination to practise. However, following recent legislative reforms, this requirement was abolished in 2024, and registration with the national dental board now suffices to obtain licensure.

Given this context, it is essential to assess whether current dental students possess adequate knowledge and self‐confidence in recognising and managing anxiety, pain and urgent or emergency medical situations.

One of the most widely used frameworks in the medical field for evaluating these dimensions is the Knowledge, Attitude and Practices model (KAPm). This model posits that clinical behaviours are influenced by underlying knowledge and attitudes. Despite its relevance, limited research has explored how effectively dental students are trained to manage medical emergencies, anxiety and pain in the clinical setting [17].

Therefore, this cross‐sectional study aims to assess the current state of Italian dental students' preparation, attitudes and perceptions regarding emergency management, anxiety and pain. Additionally, the authors explored students' perceived need for more in‐depth knowledge of these matters.

## 2 Methods

This cross‐sectional study was conducted between June 2023 and December 2023 by the Operative Dentistry Unit in the Department of Neuroscience, Reproductive and Oral Sciences of the University of Naples “Federico II” (Naples, Italy) University. The study was conducted according to the principles of the Helsinki Declaration.

### 2.1 Eligibility Criteria

The inclusion criteria were: (a) Italian dental students in their final two course years (years 5 and 6); (b) students who had already completed the anaesthesiology course and passed the exam. The exclusion criteria were: (a) foreign students; (b) students who had not completed the exams within the set time period; (c) students who had not attended the anaesthesiology course or had not yet passed the exam.

### 2.2 Sampling and Data Collection

The target population consisted of dental students in the last 2 years of the course, a total of 1652 individuals according to the data declared by the Italian Ministry of University and Research. Based on Cochran's formula (1977) assuming an alpha level of 0.05, a margin of error of 0.3 and a *Z*‐score of 1.96, it was possible to calculate a minimum sample size of 189 subjects by consulting the table developed by Bartlett, Kotrlik and Higgins for continuous variables [18]. With the aim of obtaining as representative a sample as possible, the recruitment targeted all 38 Italian faculties with the contribution of the Italian Dental Students' Association (AISO) which provided 1427 email contacts of students enrolled in the last 2 years of the course. An invitation email was sent to the students, explaining the purpose and significance of the study, along with a Google Forms link (Google LLC, California, USA) to complete a questionnaire. All questions were mandatory in the questionnaire, which was estimated to take approximately 5 min to complete. Before answering the questionnaire, participants were provided with a standard consent statement and required to give their voluntary consent to participate. The data collection process was anonymous and directly created by the Google Forms online platform. The data manager sent reminders to participants who did not respond, 2 and 4 weeks after the initial email.

### 2.3 Survey Instrument Design and Validation

The questionnaire was developed de novo, based on a review of the scientific literature and aligned with the educational objectives set by the Association for Dental Education in Europe (ADEE), particularly in the domains of anxiety and pain management and medical emergencies. Its content was shaped collaboratively by faculty members from the postgraduate Master's Programme in the Management of Anxiety, Pain and Medical Emergencies in Dentistry at the University of Naples Federico II, experts from the Italian Association of Anaesthesia in Dentistry (AIAO) and certified BLS‐D and ACLS instructors. To ensure content validity, all items were reviewed by a panel of dental educators and clinicians with expertise in pharmacology, sedation and emergency care. A pilot test was then conducted with 15 dental students from the same institution (not included in the main cohort), who provided feedback on clarity, relevance and comprehensibility. Minor wording adjustments were made accordingly.

The questionnaire consisted of two main parts: the first part collected demographic information, including gender, age, year of study and the geographical region of the university attended. The second part comprised 22 items, organised into three domains:

#### 2.3.1 Knowledge Domain

- Subdomain: Anxiety (4 items): Assesses knowledge of sedation practices, the neuroanatomy of fear, sedative pharmacology, and the mechanism of action of nitrous oxide.
- Subdomain: Pain (4 items): Covers analgesic drug classification, hypnosis in pain management and common misconceptions regarding NSAIDs.
- Subdomain: Emergencies (4 items): Focuses on BLS‐D and ACLS concepts, including chest compression‐to‐ventilation ratios, the function of automated defibrillators, recognition of pulseless electrical activity and initial response to syncope. These items were modelled on AHA training materials and reflect real‐case emergency protocols relevant to dental practice.

Each knowledge question was structured as a multiple‐choice item with five answer options, including one ‘Don't know’ option to discourage guessing. Only one answer was correct. Scoring was dichotomous: correct answers received 1 point, whereas incorrect or ‘Don't know’ answer received 0. The total score range for the knowledge domain was 0–12.

#### 2.3.2 Attitude Domain (8 Items)

Assessed students' attitudes toward sedation, opioid prescribing, emergency management, and the perceived need for continuing education. Items were rated on a 5‐point Likert scale from ‘strongly disagree’ (0) to ‘strongly agree’ (4). Two items specifically addressed continuing education needs, while six referred to confidence in applying clinical procedures in dentistry. The total score range for this section was 0–32.

#### 2.3.3 Practice Domain (2 Items)

Investigated prior participation in university courses on anxiety management and Basic Life Support (BLS). Responses were scored dichotomously: ‘Yes’ = 1; ‘No’ or ‘Don't know’ = 0. The total score range for this domain was 0–2.

**FIGURE 1:**
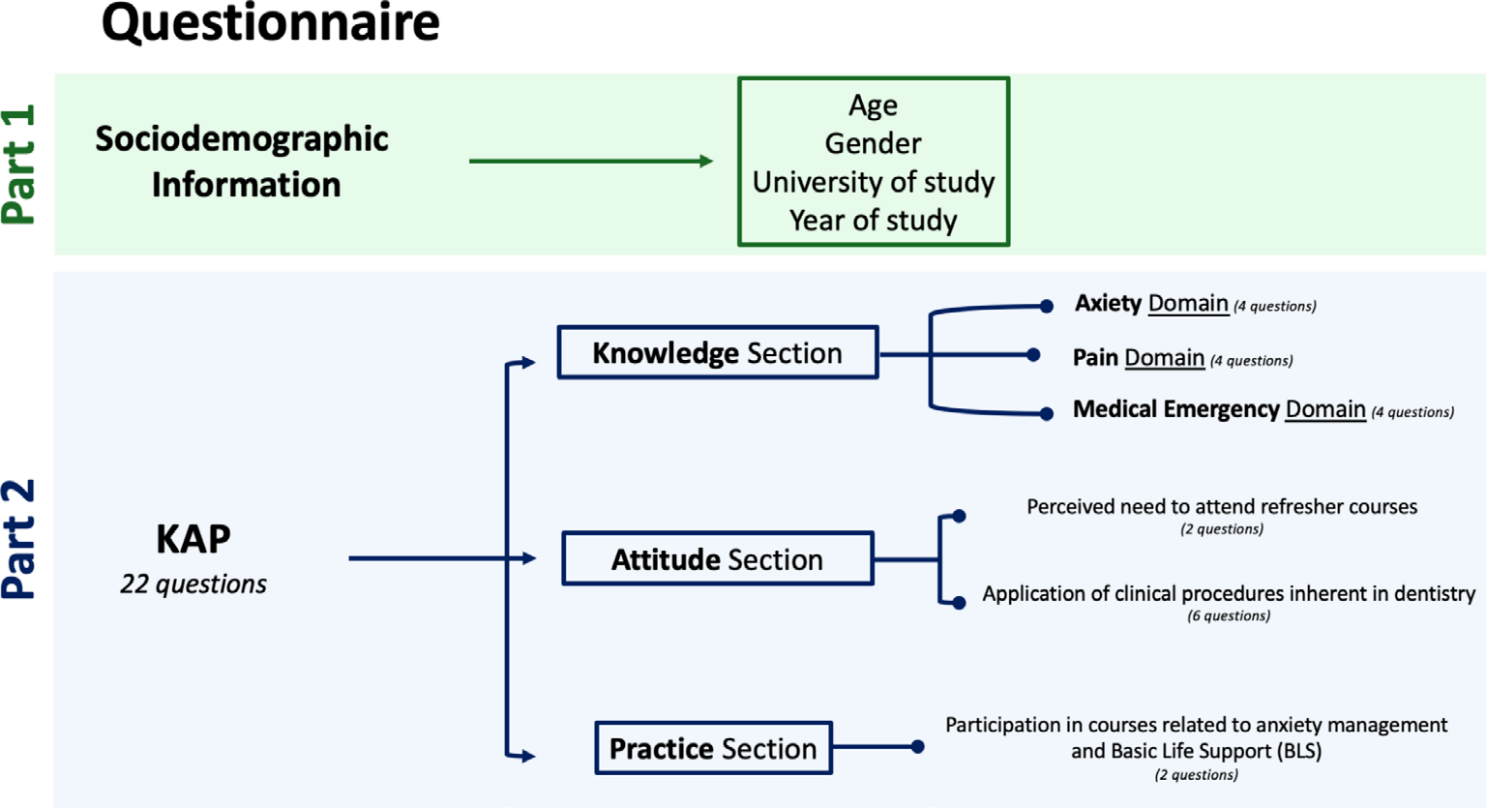
Schematic representation of the questionnaire structure.

The structure of the questionnaire is illustrated in Figure 1, while the complete version, including the correct answers, is provided in Appendix S1.

### 2.4 Statistical Analysis

An Excel data entry form and data management system were used (Microsoft Excel 2011; Windows, version 14.0.0; Microsoft Corp., Redmond, WA, USA). The Excel spreadsheet was directly generated from the online Google platform and all data were evaluated in terms of accuracy and completeness. For qualitative data, frequencies, proportions and 95% confidence intervals for proportions were calculated. In bivariate analysis, proportions were compared using two tests. The chi‐square statistic (i.e., *χ* ^2^ statistic) was calculated when no more than 20% of the cells of the contingency tables had frequencies of 5 or less and no cells had expected frequencies less than 1 [19]. If any of the observed values were less than 5, then a Fisher's exact test was performed. The threshold value decided for determining the statistical significance corresponds to a *p*‐value of 0.05 (5%). The statistician was blinded and external to the working group. Data analysis was performed with STATA/IC 16 software (StataCorp LLC, College Station, TX, USA). Furthermore, the data obtained on geographical distribution (three categories: North, Central and South) were analysed using one‐way ANOVA and Tukey's post hoc test. In addition, internal consistency of the Knowledge and Attitude sections was assessed by Cronbach's alpha calculation.

## 3 Results

**FIGURE 2:**
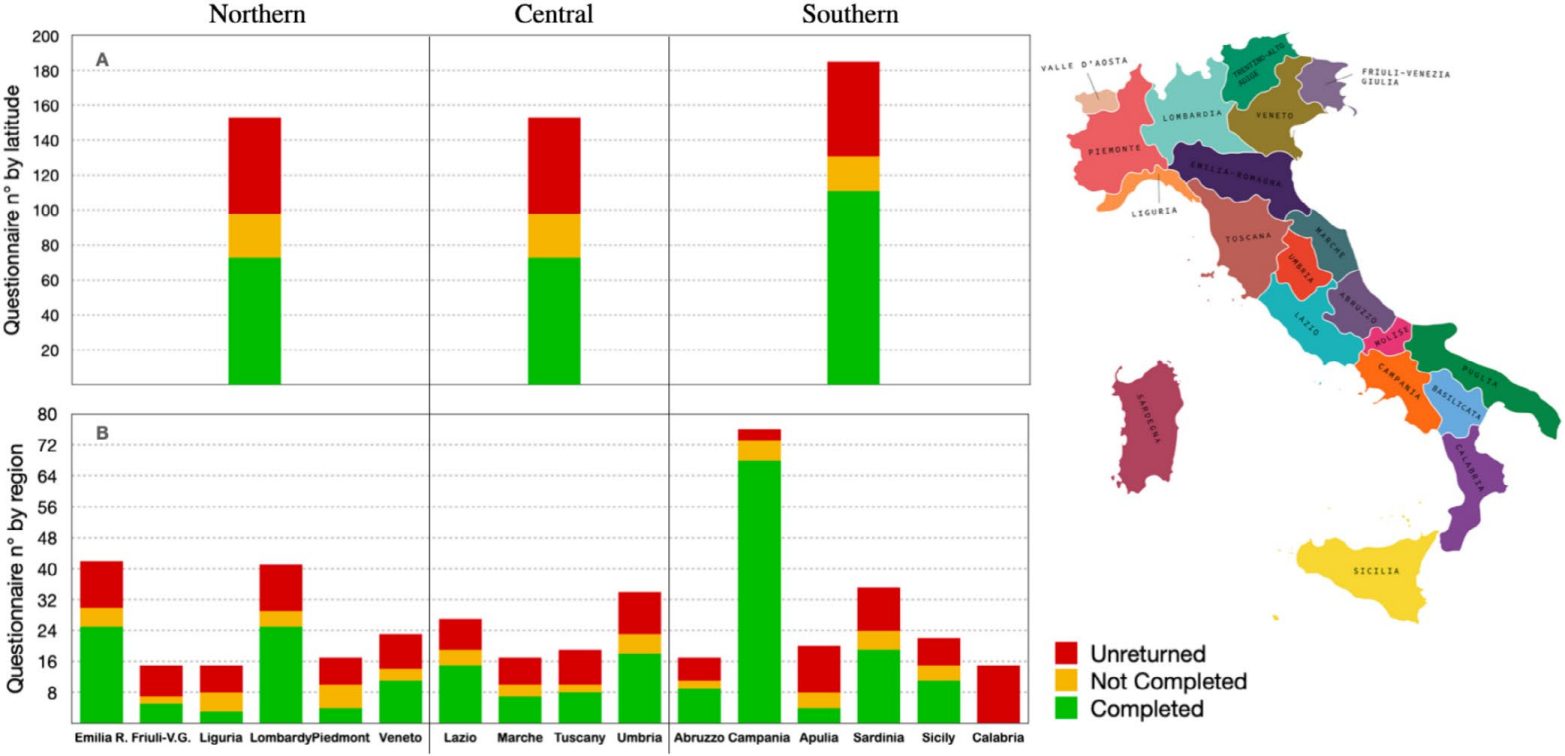
Distribution of completed, not‐completed and unreturned questionnaires by Italian region and geographical area.

Out of the 435 questionnaires distributed to students who agreed to participate, 232 were accurately completed and deemed eligible (52.16% female: 47.84% male). Of the 38 Italian universities offering dentistry courses (17 in the North, 9 in the Centre and 12 in the South of Italy), only two did not return any surveys (Lazio and Calabria). Overall, an average of 6.11 questionnaires were gathered per university site, with 75 collected in the North (32.33%, ratio 6.25), 46 in the Centre (19.83%, ratio 5.11) and 111 in the South of Italy (47.84%, ratio 9.25). The number of questionnaire invitations sent by region, along with the number of correctly completed, incomplete, or unreturned questionnaires by geographical area, is summarised in Figure 2.

### 3.1 Knowledge Section

Among the 232 completed questionnaires, the average score was 3.51 ± 1.79 points. Only 2.16% of the questionnaires received 0 points, while 70.85% fell within the range of 1 to 4 points. Additionally, 25.85% scored between 5 and 8, with just 0.86% achieving more than 8 points. Notably, none of the questionnaires scored higher than 10. From a geographical point of view, no statistically significant difference (ANOVA *p*‐value: 0.476) was observed between the North (mean 3.39 ± 1.82 points), Centre (3.35 ± 1.80 points) and South of Italy (3.66 ± 1.77 points).

#### 3.1.1 Anxiety Domain

**FIGURE 3:**
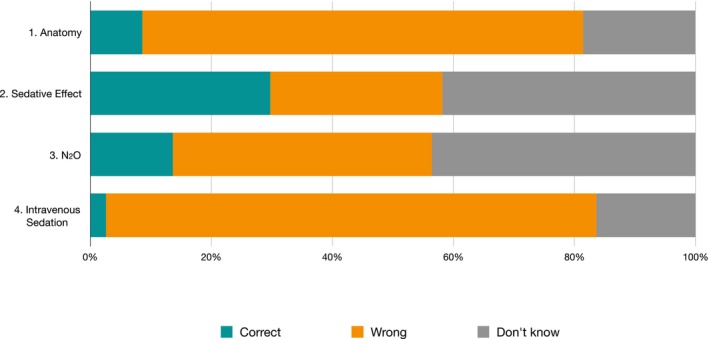
Frequency of responses regarding the anxiety domain in the knowledge section subdivided by individual question.

The frequency and distribution of responses within the anxiety domain are presented in Table 1 and Figure 3.

**TABLE 1:** Questions regarding the anxiety domain in the knowledge section. Number of correct and incorrect answers. Percentages are subdivided by region (North, Centre or South of Italy). *p*‐value is evaluated with Pearson *χ* ^2^ or Fisher's exact test.

Question numbers	Question	Correct answers		Incorrect answers		*p*
		Total population *N* (%)	Relative geographical distribution	Total population *N* (%)	Relative geographical distribution	
1 (anatomy)	Which of the following anatomical structures is responsible for the genesis of fear?	69 (29.74%)	North: 24%	163 (70.26%)	North: 76%	0.012*
			Centre: 17.39%		Centre: 82.71%	
			South: 38.73%		South: 61.27%	
2 (sedative effect)	Which of the following drugs has the lowest sedative effect?	31 (13.66%)	North: 10.67%	201 (86.64%)	North: 89.33%	0.358
			Centre: 19.57%		Centre: 80.44%	
			South: 12.61%		South: 87.39%	
3 (N_2_o)	Which of the following statements is correct regarding nitrous oxide?	6 (2.58%)	North: 0%	226 (97.42%)	North: 100%	0.162
			Centre: 2.17%		Centre: 97.83%	
			South: 4.50%		South: 95.50%	
4 (intravenous sedation)	According to your knowledge, can the dentist perform intravenous sedation manoeuvres using central nervous system depressant drugs?	20 (8.62%)	North: 6.67%	212 (91.38%)	North: 93.33%	0.712
			Centre: 10.87%		Centre: 89.13%	
			South: 9.01%		South: 90.99%	
	Total answers	126 (13.58%)		802 (86.42%)		

*Note:* * statistically significant *p*‐values (*p* < 0.05).

The mean total score was 0.54 ± 0.66 (95% CI 0.46; 0.63). A total of 125 questionnaires (53.88%) scored 0 points, while only 17 (7.33%) reached or surpassed 2 points. None achieved 4 points. Geographically, a statistically significant difference (ANOVA *p*‐value: 0.0492; Tukey *p*‐value: 0.043) was observed between the North (0.41 ± 0.59 points) and the South (0.65 ± 0.67 points). Southern Italy exhibited a significantly higher percentage of correct answers to the anatomy question than other regions (*p*‐value = 0.012). Considering the total of 928 possible answers (232 subjects multiplied by 4 questions) across all regions of Italy, the level of knowledge pertaining to anxiety was notably low, with 86.42% of answers being incorrect.

#### 3.1.2 Pain Domain

**FIGURE 4:**
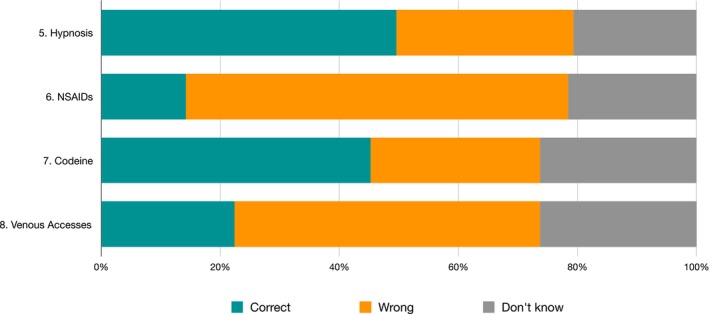
Frequency of responses regarding the pain domain in the knowledge section subdivided by individual question. NSAIDs, non‐steroidal anti‐inflammatory drugs.

Frequency and distribution of pain domain responses are shown in Table 2 and Figure 4.

**TABLE 2:** Questions regarding the pain domain in the knowledge section. Number of correct and incorrect answers. Percentages are subdivided by region (North, Centre and South of Italy). *p*‐value is evaluated with Pearson *χ* ^2^ or Fisher's exact test. NSAIDs: Non‐steroidal anti‐inflammatory drugs.

Question Numbers	Question	Correct answers		Incorrect answers		*p*
		Total Population *N* (%)	Relative geographical distribution	Total population *N* (%)	Relative geographical distribution	
5 (hypnosis)	Is it possible to achieve a complete and long‐lasting analgesia solely through hypnosis?	33 (14.22%)	North: 18.67%	199 (85.77%)	North: 81.33%	0.399
			Centre: 13.04%		Centre: 86.96%	
			South: 11.71%		South: 88.29	
6 (NSAIDs)	NSAIDs (which one of the following sentences is not correct?)	52 (22.41%)	North: 24%	180 (77.58%)	North: 76%	0.923
			Centre: 21.74%		Centre: 78.26%	
			South: 21.62%		South: 78.38%	
7 (codeine)	Codeine can be classified as:	105 (45.26%)	North: 46.67%	127 (54.74%)	North: 53.33%	0.942
			Centre: 45.65%		Centre: 54.35%	
			South: 44.14%		South: 55.86%	
8 (venous accesses)	The dentist can find venous accesses for the administration of drugs?	115 (49.57%)	North: 49.33%	117 (50.43%)	North: 50.67%	0.857
			Centre: 47.83%		Centre: 52.17%	
			South: 52.25%		South: 47.75%	
	Total answers	305 (32.87%)		623 (67.13%)		

Considering the total number of answers, the level of knowledge relating to Pain was low, with 86.42% of answers incorrect. No statistical difference was found between the three geographical areas.

#### 3.1.3 Medical Emergency Domain

**FIGURE 5:**
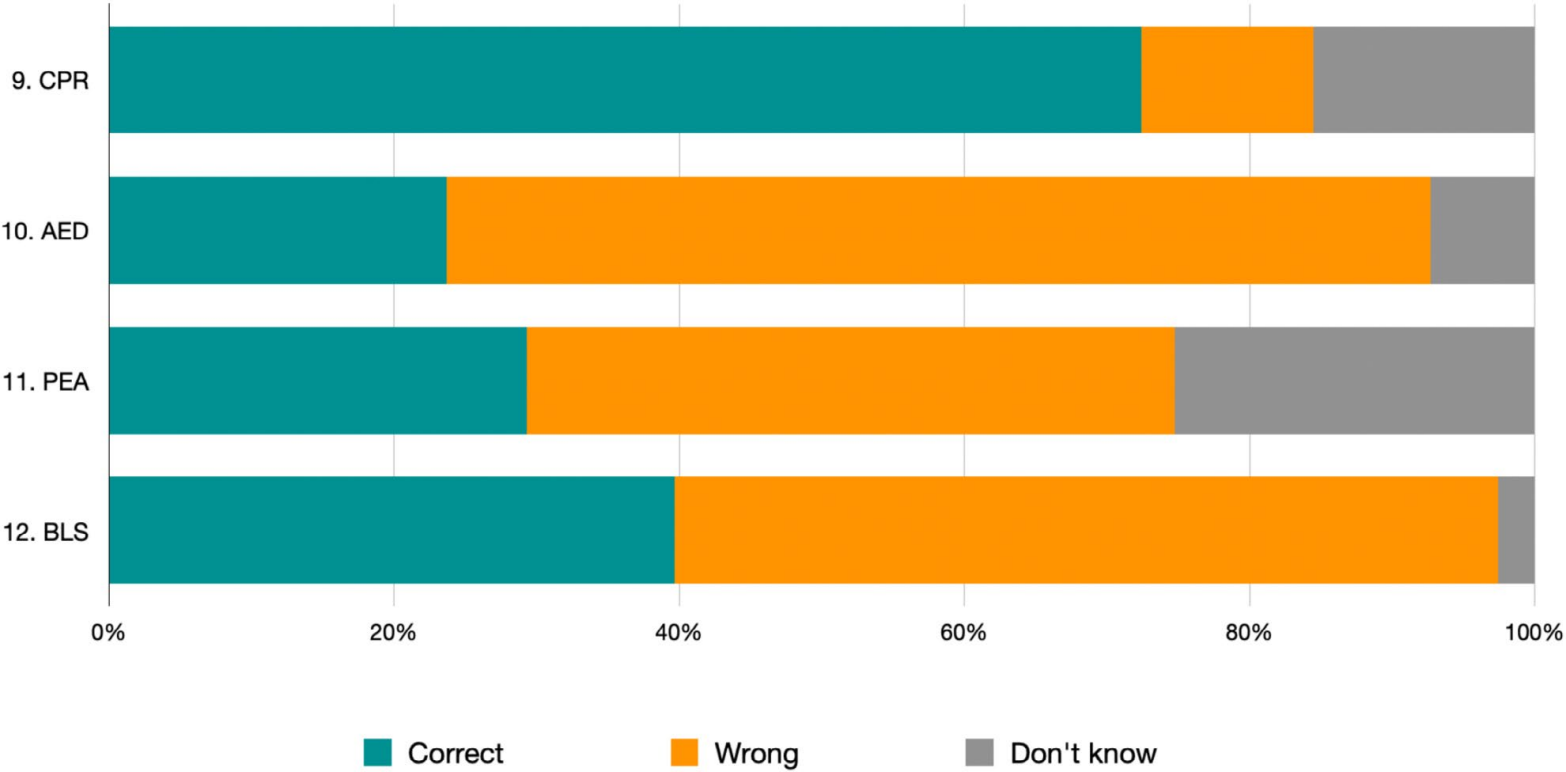
Frequency of responses regarding the medical emergency domain in the knowledge section divided by individual question. AED, automatic external defibrillator; BLS, basic life support; CPR, cardiopulmonary resuscitation; PEA, pulseless electrical activity.

Frequency and distribution of medical emergency domain responses are shown in Table 3 and Figure 5. The domain relating to medical emergency was the one with the best result, with an overall 41.27% of correct answers. No statistical difference was found between the three geographical areas.

**TABLE 3:** Questions regarding the medical emergency domain in the knowledge section. Number of correct and incorrect answers. Percentages are subdivided by region (North, Centre and South of Italy). *p*‐value is evaluated with Pearson *χ* ^2^ or Fisher's exact test.

Question numbers	Question	Correct answers		Incorrect answers		*p*
		Total population *N* (%)	Relative geographical distribution	Total population *N* (%)	Relative geographical distribution	
9 (CPR)	In the event of cardiac arrest in adults, the ratio between chest compressions and ventilations that has demonstrated greater effectiveness in maintaining a perfusion useful for supporting vital functions is?	168 (72.41%)	North: 74.67%	64 (27.59%)	North: 25.33%	0.472
			Centre: 65.22%		Centre: 34.78%	
			South: 73.87%		South: 26.13%	
10 (AED)	The use of the semi‐automatic defibrillator (AED) allows you to?	55 (23.71%)	North: 24%	177 (76.30%)	North: 76%	0.890
			Centre: 26.09%		Centre: 73.91%	
			South: 22.52%		South: 77.48%	
11 (PEA)	In case of pulseless electrical activity (PEA)?	68 (29.31%)	North: 20%	164 (70.69%)	North: 80%	0.061
			Centre: 28.26%		Centre: 71.74%	
			South: 36.04%		South: 63.96%	
12 (BLS)	If a patient suddenly feels unwell and loses consciousness, what is the first action to take?	92 (39.66%)	North: 40%	140 (60.34%)	North: 60%	0.914
			Centre: 36.96%		Centre: 63.04%	
			South: 40.54%		South: 59.46%	
	Total answers	383 (41.27%)		545 (58.73%)		

Abbreviations: AED, automatic external defibrillator; BLS, basic life support; CPR, cardiopulmonary resuscitation; PEA, pulseless electrical activity.

### 3.2 Attitude Section

**FIGURE 6:**
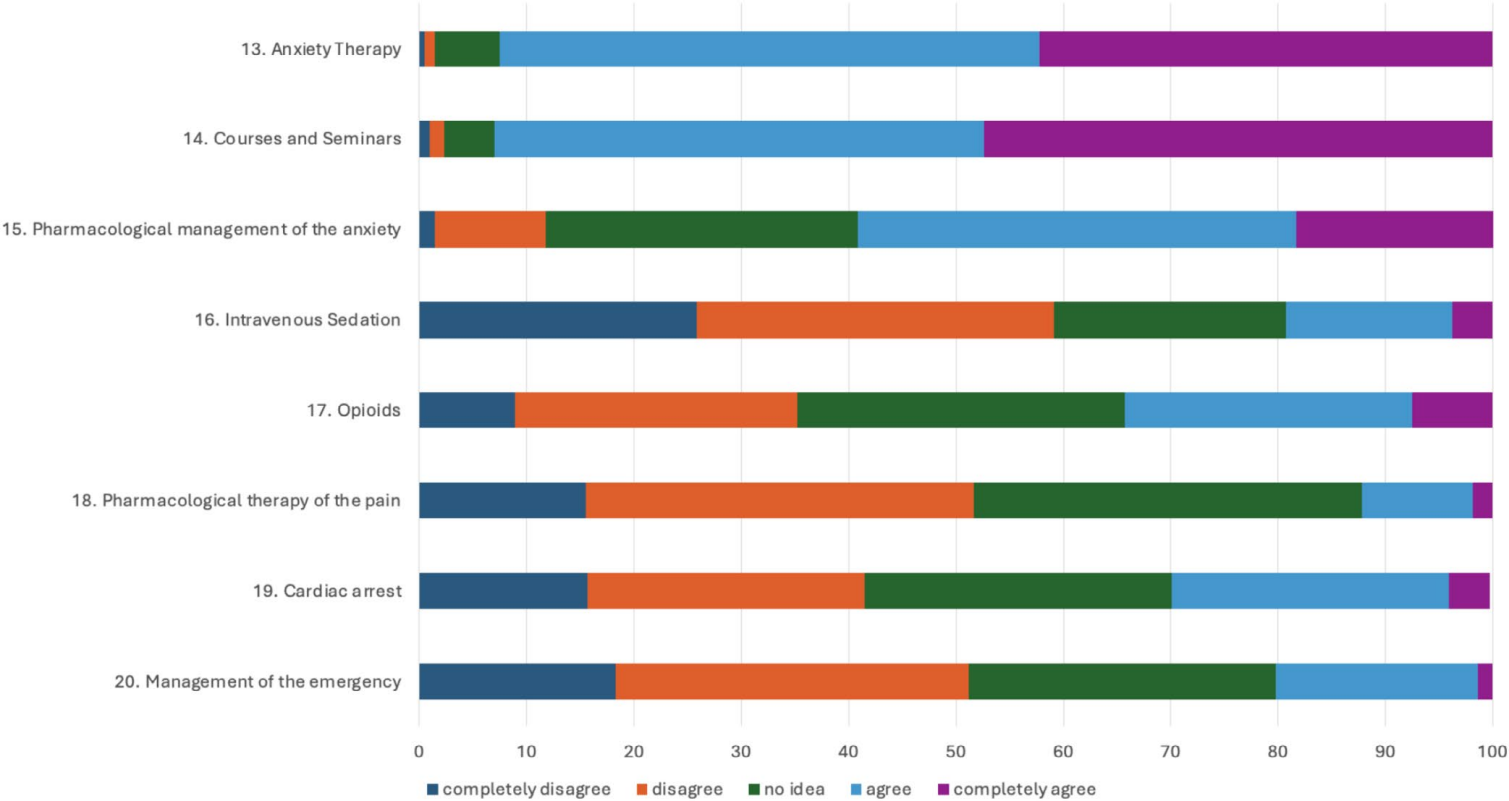
Frequency of responses regarding attitude section subdivided by individual question.

The questions from the Attitude and Perception section are shown in Table 4 and Figure 6.

**TABLE 4:** Questions in the attitude and perception section.

Question numbers	Questions
13 (Anxiety therapy)	I need more information about anxiety therapy
14 (Courses and seminars)	I am willing to attend courses and seminars on sedation and pain management
15 (Pharmacological management of anxiety)	I believe that the pharmacological management of anxious patients undergoing dental procedures is the responsibility of the dentist
16 (Intravenous sedation)	I am/feel comfortable establishing peripheral venous access for drug administration
17 (Opioids)	I am/will be comfortable prescribing opioid medications
18 (Pharmacological therapy of the pain)	I believe my training in pharmacological pain management is adequate.
19 (Cardiac arrest)	Based on the knowledge acquired, I believe I am capable of managing the initial response to a patient in cardiac arrest
20 (Emergency management)	I believe dentists are adequately trained to handle emergencies that may arise in a dental office

This section was made up of eight questions: two concerning the perceived need to attend refresher courses (questions 13 and 14) and six concerning the application of clinical procedures inherent in dentistry (questions 15–20).

Out of 32 obtainable points, the mean total score was 17.45 ± 3.68 (CI 16.95; 17.94). None of the tests reached 32 points or fell below 9 points. 14.1% of students (30 out of 232) scored from 9 to 13; 48.36% (103 out of 232) scored from 14 to 18; 33.33% (71 out of 232) scored from 19 to 23 and 4.23% (9 out of 232) scored from 23 to 31. No statistical difference was found between the three geographical areas.

Questions 13 (*I need more information about anxiety therapy*) and 14 (*I am inclined to participate in courses and seminars on sedation and pain therapy*) in Table 4 regard the perceived need to attend refresher courses. Out of 8 total achievable points, the mean score was 6.70 ± 1.14 (CI 6.55; 6.85). Only 9.39% of students obtained a score of less than 6, while 90.61% obtained at least 6 points. No statistical difference was observed between North, Centre and South (ANOVA *p*‐value: 0.6150).

For the other questions (15–20), regarding attitude in performing operating procedures, out of 24 possible points, the mean score was 10.75 ± 3.67. 95.31% achieved a total of no more than 16 points. Only 4.69% scored 17 to 23 points. No significant difference was found between North, Centre and South.

### 3.3 Practice Section

**FIGURE 7:**
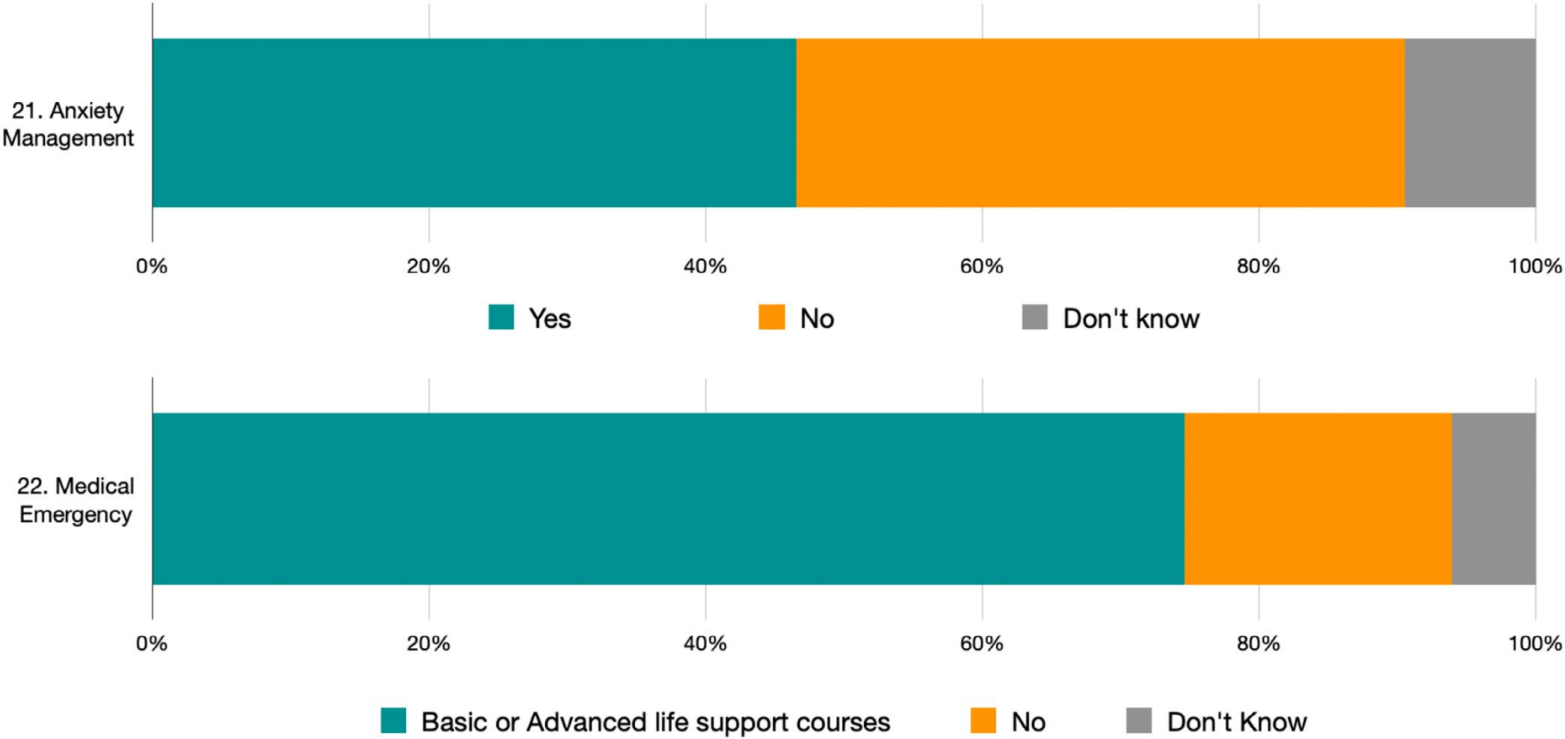
Frequency of responses in practice section subdivided by individual question.

The questions of the Practice section regarding participation in courses are presented in Table 5 and Figure 7.

**TABLE 5:** Questions of the practice section.

Question numbers	Question
21 (Anxiety management)	During your undergraduate studies, did you attend any course where you developed skills in identifying and managing dental anxiety?
22 (Medical emergency)	During your undergraduate studies, did you attend any course where you developed skills in performing basic life support (BLS) and/or advanced life support (ALS = drug administration and ECG interpretation in emergencies)?

53.45% of students state that they have never taken or do not remember having taken courses for anxiety. Only 10.8% answered ‘yes’. Regarding emergencies, 25.43% have never attended or do not remember having attended any Basic or Advanced Life Support courses. Out of a maximum achievable score of 4 points, the overall average was 1.86 ± 1.15, with no difference between North, Centre and South of Italy (ANOVA *p*‐value: 0.2673).

Internal consistency of the Knowledge section was assessed using Cronbach's alpha, resulting in a value of 0.386. This lower alpha value reflects the multidimensional nature of the questionnaire, which included diverse items covering anxiety management, pain control and emergency procedures.

The internal consistency of the Attitude section was assessed using Cronbach's alpha, resulting in a value of 0.76, indicating good reliability.

## 4 Discussion

In the dental student's course, the disciplines that provide the knowledge and skills necessary to identify anxious patients, treat pain and prevent/treat possible medical emergencies/urgent situations that may occur in daily clinical practice must be comprehensively addressed. There are no studies in the current literature that take into consideration the knowledge, attitude and practice of dental students on these issues. Overall, the results of this study indicated a geographical homogeneity in the identification, management, and prevention of anxiety, pain and medical emergencies in the students examined.

The results, however, highlighted significant knowledge gaps in all three domains (anxiety, pain and medical emergencies).

This limited knowledge base may be explained by how these topics are addressed within the Italian dental curriculum. Issues such as anxiety management, conscious sedation, pharmacological pain control and medical emergency response are not typically taught as independent subjects. Rather, they are distributed across various broader courses, including pharmacology, paediatric dentistry, oral surgery and anaesthesiology. As a result, coverage is often fragmented and lacks the depth required for clinical confidence. Furthermore, there is no national standardised system for assessing the acquisition of these competencies. Each university independently structures its curriculum and determines student progression, which is usually based on the accumulation of course credits and oral or written examinations administered by individual instructors. This decentralisation may lead to inconsistencies in training and evaluation across institutions, especially in interdisciplinary and clinically applied domains such as those investigated in this study.

Notably, a dedicated teaching chair in the subject ‘Anaesthesia and Sedatives Used in Dentistry’, formally assigned to graduates in Dentistry (class MED/28), is absent in all Italian universities, despite being mandated by the European Directive 78/687/EEC of the Council of 25 July 1978. This discipline is explicitly intended to cover topics such as sedation, pain management, and medical emergencies in dentistry, and should be taught by professionals with specific dentistry training and recognised expertise in these areas. The absence of this formal structure contributes to the lack of consistent, competence‐based education in these critical aspects of dental practice.

A broader concern about inadequate preparedness for medical emergencies among dental professionals has also been documented in other European contexts. Recent evidence from Eastern Europe has further confirmed that many practising dentists reported insufficient training and low confidence in handling urgent medical scenarios, further emphasising the need for early and consistent education on this topic during undergraduate training [20].

Anxiety emerged as the least understood domain, with only 13.6% of responses correct. The lowest‐performing items were related to the pharmacology of nitrous oxide (2.6% correct) and intravenous sedation (8.6%), indicating that students lack both theoretical understanding and regulatory awareness regarding sedation procedures. A high proportion of ‘I don't know’ responses suggest uncertainty possibly due to the fragmented way these topics are taught, often integrated superficially within broader courses. This lack of clarity is consistent with the finding that over 50% of students reported never attending a specific course on anxiety management. Notably, more than 90% expressed willingness to attend further training in this area.

Knowledge of pain management was higher, but still limited (32.9% overall). Only 22.4% of respondents correctly identified a false statement about NSAIDs, and 14.2% correctly answered a question about hypnosis as a sole analgesic method. These gaps may stem from insufficient pharmacological training and lack of integration between theory and clinical practice. Nearly half of the students (49.6%) knew that dentists are endorsed to perform intravenous drug administration, but the other half were uncertain or misinformed, reflecting poor clarity in the legal and clinical competencies taught at undergraduate level.

These findings are consistent with broader European trends. A study by Jaldin et al. [21] revealed significant knowledge gaps and attitudinal inconsistencies among dental and dental hygiene students regarding paediatric pain management. The most critical deficiencies were found in students' understanding of pain perception in young children, the importance of parental presence during painful procedures and clinical pain assessment. The authors attributed these shortcomings to inconsistencies and outdated content in dental curricula, which often fail to integrate interdisciplinary, evidence‐based recommendations such as those promoted by the International Association for the Study of Pain (IASP) [22]. These observations further support the need for harmonised training programmes that better prepare future dental professionals to manage pain in various clinical contexts.

Emergency management was the best‐performing domain (41.3% correct answers). While over 70% of students correctly answered the CPR‐related question, knowledge of advanced concepts, such as the role of defibrillators (23.7%) and pulseless electrical activity (29.3%), was low. Moreover, only 39.7% knew the correct first action to take in the event of a sudden loss of consciousness. These findings suggest basic theoretical preparation but insufficient consolidation through simulation or clinical training. Despite more than 70% of respondents having attended emergency‐related courses, only a minority felt confident on managing cardiac arrest scenarios, pointing to the limited practical impact of current educational programmes.

Costa et al. [23] have shown that almost all dentists interviewed provide treatment to children with dental anxiety/fear or behavioural management problems, and 91.2% do not systematically diagnose these conditions. Slightly better, but not encouraging, were the results relating to the treatment of pain and medical emergencies.

The Joint Commission Statement on Pain Management indicates that the decision‐making process for choosing which painkiller/anti‐inflammatory drugs to use must be based on a patient‐centred approach [24]. It is, therefore, essential to know drugs and administration routes as well as the clinical and psycho‐emotional conditions of the patient. In Italy, it seems that almost 30% of students do not believe that the dentist is able to obtain venous access for the parenteral administration of drugs. Furthermore, knowledge of the mechanisms of action of NSAIDs was not satisfactory, and only 45% of those interviewed considered opioids as centrally acting drugs of second choice. Stafuzza et al. [25], in a sample of 100 dentists, found that most (87%) were trained in Basic Life Support (BLS), but only 43% considered themselves capable of providing first aid and performing the necessary procedures.

Also, Laurent et al. [17] highlighted that 53% of the subjects interviewed considered themselves totally or sufficiently qualified to carry out basic Cardiopulmonary Resuscitation (CPR). In the same study, however, the authors simulated emergency situations by testing the real practical skills of the students, who were found to be incapable of competently managing a cardiac arrest. Their results partially align with those of the current study, as they show that three‐quarters of the subjects examined had a fair knowledge of the application principles of BLS procedures and did not know the importance of alerting the emergency system and the rationale of using a defibrillator. In addition, approximately 70% did not appear to know the main algorithms of advanced life support procedures using life‐saving drugs.

Unlike what was reported by Laurent F. et al. [17], in this study only 30% or less of the Italian students participating in the survey stated that they felt capable of dealing with the initial management of a patient in cardiac arrest, and over 50% did not consider dentists competent in medical emergency management. These data demonstrate a greater awareness of the skills acquired. Considering that over 70% have attended university courses on medical emergency management, the data reported suggest the need to make training courses on the topic more effective. It should be noted that approximately one‐quarter of the students stated that they had never attended a course on medical emergencies during their degree course. These percentages are much higher than those reported in the literature [25]. These findings are consistent with broader trends observed among Italian dental professionals. A recent large‐scale survey involving over 6800 dentists reported that although 65.2% had encountered at least one medical emergency during their career, only a minority had received formal training during their academic education. Notably, 98.6% of respondents expressed a strong desire for more theoretical‐practical institutional training and the establishment of a national emergency registry, highlighting widespread recognition of current educational deficiencies [26].

Concerning the incidence of medical complications in dental clinical practice, the most frequent ones are syncope, followed by epileptic seizures and metabolic complications (diabetes) [27]. Fear and/or anxiety are exacerbating factors common to many of these. Insufficient basic preparation, associated with poor clinical experience, predisposes pre‐doctoral students to have a higher incidence of complications than more professionally mature personnel [28].

Nonetheless, in recent years, there has been a growing interest within the Italian dental community in the management of anxiety, pain and medical emergencies. This is evidenced by the establishment of two dedicated scientific societies—the Italian Association of Anaesthesia in Dentistry and the Italian Association of Sedation Dentists—which actively promote continuing education programmes and postgraduate training on these critical topics. These developments reflect an increasing awareness of the need to integrate pharmacological knowledge, sedation techniques, and emergency protocols into everyday clinical dental practice.

The considerable demand for postgraduate training also appears to confirm the existence of educational gaps that were not adequately addressed during undergraduate dental education.

Finally, despite the valuable insights provided, this study has some limitations that should be acknowledged: the internal consistency of the *Knowledge* section was low (Cronbach's alpha = 0.386), likely due to the heterogeneity of content across different domains. The self‐reported nature of the data may have introduced reporting bias. The response rate was moderate, possibly affected by the voluntary participation, timing and email‐based recruitment. The sampling was non‐random and limited to students who had passed the anaesthesiology course, which may reduce generalisability. Additionally, the questionnaire, although expert‐informed and pilot‐tested, has not yet been psychometrically validated on a large scale. Finally, the cross‐sectional design prevents any assessment of changes over time or causal relationships.

## 5 Conclusion

This study highlights the need for curricular reform at the undergraduate level to address significant gaps in the management of anxiety, pain and medical emergencies. Implementing a standardised, competency‐based national curriculum would help ensure consistent clinical preparedness among dental graduates and improve patient safety and care quality.

## Conflicts of Interest

The authors declare no conflicts of interest.

## Supporting information


Data S1.

## Data Availability

The data that support the findings of this study are available on request from the corresponding author. The data are not publicly available due to privacy or ethical restrictions.
