# Supplementary material for: Knowledge, Attitude and Practice in Anxiety, Pain and Medical Emergency Management: A National Survey on 232 Italian Dental Students
Source: Eur J Dent Educ. 2025 Jul 11;30(2):661–72. doi: 10.1111/eje.70012 (PMC13090433; doi:10.1111/eje.70012)
Supplement: Supplementary file 1 — Data S1. [file EJE-30-661-s001.docx]

**Domain: KNOWLEDGE**

**Subdomain: ANXIETY**

1. Which of the following anatomical structures IS RESPONSIBLE for the genesis of fear?
   **a) Temporal lobe***
   b) Parietal lobe
   c) Occipital lobe
   d) Frontal lobe
   e) I don’t know
2. Which of the following drugs has THE LOWEST sedative effect?
   **a) Diazepam***
   b) Triazolam
   c) Midazolam
   d) Propofol
   e) I don’t know
3. Which of the following statements IS CORRECT regarding nitrous oxide?
   **a) It promotes endorphin release***
   b) It has a rapid onset of action, noticeable after 10 minutes
   c) It acts on GABA receptors inducing moderate sedation
   d) It is a sedative drug and has no analgesic effect
   e) I don’t know
4. According to your knowledge, can the dentist perform intravenous sedation procedures using central nervous system depressant drugs?
   a) No
   **b) Yes***
   c) Yes, but only in collaboration with anaesthesiologists
   d) Not in an outpatient setting, only in a hospital setting
   e) I don’t know

**Subdomain: PAIN**

1. Is it POSSIBLE to achieve a complete and long-lasting analgesia solely through hypnosis?
   a) No
   **b) Yes***
   c) Hypnosis has no indication in dentistry
   d) Hypnosis is not a recognized therapeutic strategy
   e) I don’t know
2. Which one of the following sentences IS NOT correct?
   **a) NSAIDs inhibit the synthesis of arachidonic acid and prostaglandins***
   b) COX-2 inhibitors (coxibs) are associated with high cardiovascular risk
   c) Some NSAIDs act centrally as well as peripherally
   d) I don’t know
   e) Oxicams are NSAIDs with high gastric toxicity
3. Codeine can be classified as:
   b) A first-line central-acting analgesic
   **a) A second-line central-acting analgesic***
   c) A first-line peripheral-acting analgesic
   d) A second-line peripheral-acting analgesic
   e) I don’t know
4. Based on your academic training, is a dentist allowed to gain venous access for drug administration?
   a) No, never
   **b) Yes, always***
   c) Yes, but only in collaboration with anaesthesiologists
   d) Not in an outpatient setting, only in a hospital setting
   e) I don’t know

**Subdomain: EMERGENCIES**

1. In the event of cardiac arrest in adults, the ratio between chest compressions and ventilations that has demonstrated greater effectiveness in maintaining a perfusion useful for supporting vital functions is?
   **a) 30/2***
   b) 25/3
   c) 25/2
   d) 35/3
   e) I don’t know
2. The use of a semi-automatic defibrillator (AED) allows you to:
   **a) Restoration of electrical activity that was not producing cardiac output***
   b) Reactivation of electrical activity in an heart that has arrested
   c) Cardioversion
   d) Increased contractile capacity of the ventricles
   e) I don’t know
3. In the case of pulseless electrical activity (PEA):
   **a) The first-line drug is adrenaline***
   b) The first-line drug is adrenaline if the defibrillator was not effective
   c) The first-line drug is amiodarone
   d) A semi-automatic defibrillator should be activated as soon as possible
   e) I don’t know
4. If a patient suddenly feels unwell and loses consciousness, what is the first action to take?
   **a) Call for help***
   b) Start ventilation procedures
   c) Check carotid pulse
   d) Look for a defibrillator
   e) I don’t know

**Domain: ATTITUDE**

1. I feel I need more information on anxiety management.
   a) Strongly agree
   b) Agree
   c) Neither agree nor disagree
   d) Disagree
   e) Strongly disagree
2. I am willing to attend courses and seminars on sedation and pain management.
   a) Strongly agree
   b) Agree
   c) Neither agree nor disagree
   d) Disagree
   e) Strongly disagree
3. I believe that the pharmacological management of anxious patients undergoing dental procedures is the responsibility of the dentist.
   a) Strongly agree
   b) Agree
   c) Neither agree nor disagree
   d) Disagree
   e) Strongly disagree
4. I am/feel comfortable establishing peripheral venous access for drug administration.
   a) Strongly agree
   b) Agree
   c) Neither agree nor disagree
   d) Disagree
   e) Strongly disagree
5. I am/feel comfortable prescribing opioid medications.
   a) Strongly agree
   b) Agree
   c) Neither agree nor disagree
   d) Disagree
   e) Strongly disagree
6. I believe my training in pharmacological pain management is adequate.
   a) Strongly agree
   b) Agree
   c) Neither agree nor disagree
   d) Disagree
   e) Strongly disagree
7. Based on the knowledge acquired, I believe I am capable of managing the initial response to a patient in cardiac arrest.
   a) Strongly agree
   b) Agree
   c) Neither agree nor disagree
   d) Disagree
   e) Strongly disagree
8. I believe dentists are adequately trained to handle emergencies that may arise in a dental office.
   a) Strongly agree
   b) Agree
   c) Neither agree nor disagree
   d) Disagree
   e) Strongly disagree

**Domain: PRACTICE**

1. During your undergraduate studies, did you attend any course where you developed skills in identifying and managing dental anxiety?
   a) Yes
   b) No
   c) I don’t know
2. During your undergraduate studies, did you attend any course where you developed skills in performing basic life support (BLS) and/or advanced life support (ALS = drug administration and ECG interpretation in emergencies)?
   a) Yes
   b) No
   c) I don’t know
